# Staphylococcus Scalded Skin Syndrome-Induced Thrombosis Leading to Free Flap Complications: A Case Report and Review

**DOI:** 10.7759/cureus.58173

**Published:** 2024-04-13

**Authors:** Muireann Keating, Li Jie Helena Yoo, Billy Lane-O'Neill, Tom Moran, Fionnula Ni Ainle, Fergal J Moloney, Shirley Potter

**Affiliations:** 1 Department of Plastic and Reconstructive Surgery, Mater Misericordiae University Hospital, Dublin, IRL; 2 Department of Dermatology, Mater Misericordiae University Hospital, Dublin, IRL; 3 Department of Otolaryngology, Head and Neck Surgery, Mater Misericordiae University Hospital, Dublin, IRL; 4 Department of Medicine, University College Dublin, Dublin, IRL; 5 Department of Hematology, Mater Misericordiae University Hospital, Dublin, IRL

**Keywords:** hypercoagulable, thrombophilia, microsurgery, staphylococcus infection, free-flap failure

## Abstract

Staphylococcal scalded skin syndrome (SSSS) is a clinical term used for a spectrum of blistering skin conditions induced by the epidermolytic toxins of the *Staphylococcus aureus* bacteria. The complications of SSSS include thrombosis; however, the pathophysiology of this is still poorly understood. We present a case of free anterolateral thigh (ALT) flap failure in a patient as a result of widespread flap thrombosis associated with staphylococcal scalded skin syndrome (SSSS). This is the first reported case of free flap failure associated with SSSS. Free flap failure due to acquired prothrombotic conditions, such as infection, is a rare and potentially under-reported phenomenon. This article aims to further explore the role of both thrombophilias and provoked thrombotic events in free flap failure. A review of the literature will also be presented, and cases of free flap failure in patients with infection-induced vascular complications will be summarised.

## Introduction

Free flaps are currently considered the gold standard in head and neck reconstruction, with reported success rates of up to 96% [1]. Free flap failure leads to significant functional and aesthetic morbidity, as well as additional operative procedures, prolonged hospital stay and overall increased healthcare costs. Arterial and venous thromboses are the most common causes of free flap failure [2]. Identifying the cause of such thrombosis can be difficult and is often multi-factorial. Technical error, surgeon experience and type of anastomosis are all potential causes, but there can often be patient factors contributing to thrombosis. Inherited and acquired thrombophilia are recognised rare aetiologies for free flap failure and thrombosis [3].

Thrombophilia is defined as a predisposition to form clots inappropriately [4]. This predisposition can be due to genetic factors, acquired changes in the clotting process or most commonly a combination of both [5]. Hereditary thrombophilias result when an inherited factor, such as antithrombin, is defective, resulting in a predisposition to clotting.

The presence of any infection is an independent risk factor for venous thromboembolism [6]. The role of infection-mediated prothrombotic states was highlighted during the COVID-19 pandemic [7]. However, such thrombotic events can occur with a wide variety of systemic infections. Thrombotic events in COVID-19 patients were most often seen with more severe disease and often occurred even when patients were on prophylactic anticoagulation. Thrombotic events observed in patients with COVID-19 included cases of free flap failure [8,9]. There is limited literature published about the effects of systemic infection on vascular events in free flap surgery. We report a case of free flap failure in a patient with hypercoagulability due to the systemic effects of a post-operative SSSS infection.

## Case presentation

The patient was a 51-year-old male, American Society of Anesthesiologists (ASA) grade 2, who was admitted for elective resection of an invasive well-differentiated adenocarcinoma of his left hard palate, with a free anterolateral thigh (ALT) flap reconstruction. The patient had a tumour of the hard palate extending into the roof of his maxillary sinus, with a large left jugulodigastric node identified on imaging. The patient underwent the standard staging workup and planning for surgery. No significant contraindications to surgery were found on pre-operative anaesthetic assessment.

The surgery proceeded without any intraoperative complications. The patient had resection of the tumour, with a left neck dissection of levels 2a, 2b, 3 and 4 and maxillectomy as part of his tumour clearance. Frozen sections of the left jugulodigastric node and margins were taken at the time of surgery. The frozen section tissue margins from the floor of the maxillary sinus, soft palate and septum were all reported as negative prior to proceeding with immediate reconstruction. For the ALT reconstruction, three perforators were identified in the right thigh and the skin paddle (6x10cm) was raised on the most distal perforator. Dissection continued down to the descending branch of the lateral circumflex femoral artery, and the pedicle was traced to its most proximal point in the leg. The skin paddle and fascia was inset into the maxillectomy defect, and the pedicle tunnelled through the oral cavity to the left neck, where it was anastomosed end-to-end to an anterior branch of the left internal jugular vein (4mm venous coupler) and the left facial artery (hand sewn with 9/0 nylon). A second anonymous vein from the flap was anatomised to a smaller recipient vein using a 3mm coupler. The total ischaemic time was 1 hour and 30 minutes. Flap raise, inset and microvascular anastomoses were uneventful. The total recorded surgical time including resection and flap reconstruction was 8 hours and 44 minutes. The patient was brought to the High Dependency Unit post-operatively and underwent hourly flap observations with no issues for the first 24 hours.

**Figure 1:**
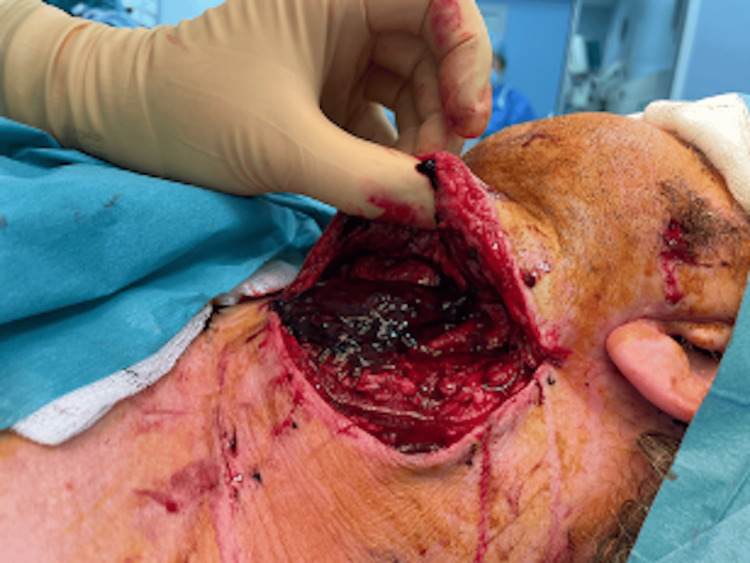
Intraoperative photograph of haematoma Haematoma in the patient's neck identified on the first return to the theatre.

Twenty-four hours post-operatively the flap was noted to be congested. The patient was immediately brought back to the theatre, and on opening the neck, 100ml of a haematoma was noted (Figure 1). There was thrombosis of both veins and a pinhole leak in the artery at the anastomosis site. The artery was repaired with a single 9-0 nylon suture. Both venous anastomoses were revised, one to a branch of the internal jugular vein using a 4mm venous coupler. The proximal external jugular vein was used as vein graft for the second vein, side to side onto the internal jugular with 9/0 nylon. A bolus of intravenous heparin was given intraoperatively. Good flow and Doppler were recorded on a table and immediately post-operatively and the patient returned to the High Dependency Unit for ongoing hourly flap observation.

Twenty-four hours later, the flap was noted to be congested again. The patient was brought back to the theatre for an attempted salvage. This time there was no haematoma or collection within the neck. Both veins were thrombosed. The artery was patent, but there was no flow distal to the anastomosis site due to venous engorgement. On excision of the venous anastomosis at the coupler sites, there were multiple clots. The arterial anastomosis was patent on excision. The artery was revised with 9-0 nylon; however, no venous flow was achieved. The artery and vein were clipped at the donor and recipient site, and the flap was left in situ.

During the immediate post-operative period, the patient complained of a painful erythematous rash on both upper limbs and the Dermatology team were consulted. The patient was distressed, describing the pain as the worst he had ever experienced. The pain was out of proportion as it only affected 10% of his body surface area. The rash was subtly noted on his elbows but was extremely tender, and Nikolsky’s sign was positive. No mucosal involvement was noted. Toxic epidermal necrolysis was the top differential, and this highlighted the urgent need for a definite diagnosis. Biopsies, including frozen sections, showed subcorneal splitting with neutrophilic infiltrates suggesting staphylococcal scalded skin syndrome (SSSS) or bullous impetigo. Swabs for culture and sensitivity were taken from blood, central line, and sputum including screening for methicillin-resistant *Staphylococcus aureus* (MRSA). Intravenous vancomycin was commenced for broad-spectrum staphylococcal cover in addition to Co-amoxiclav which had been started empirically post-surgery. On further questioning, it was discovered that the patient had an area of dermatitis on his right hand which he had been scratching aggressively prior to his surgery. This was the likely source of infection.

**Figure 2:**
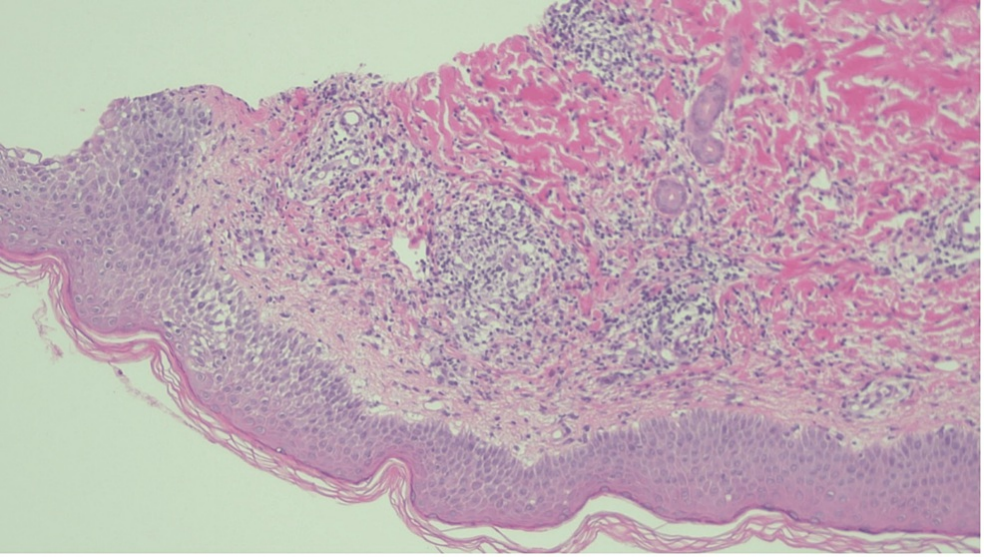
Medium power view (10x) Histology slide at medium power (10x) showing subcorneal and intraepidermal vesicles containing neutrophils, consistent with SSSS. SSSS: staphylococcal scalded skin syndrome.

**Figure 3:**
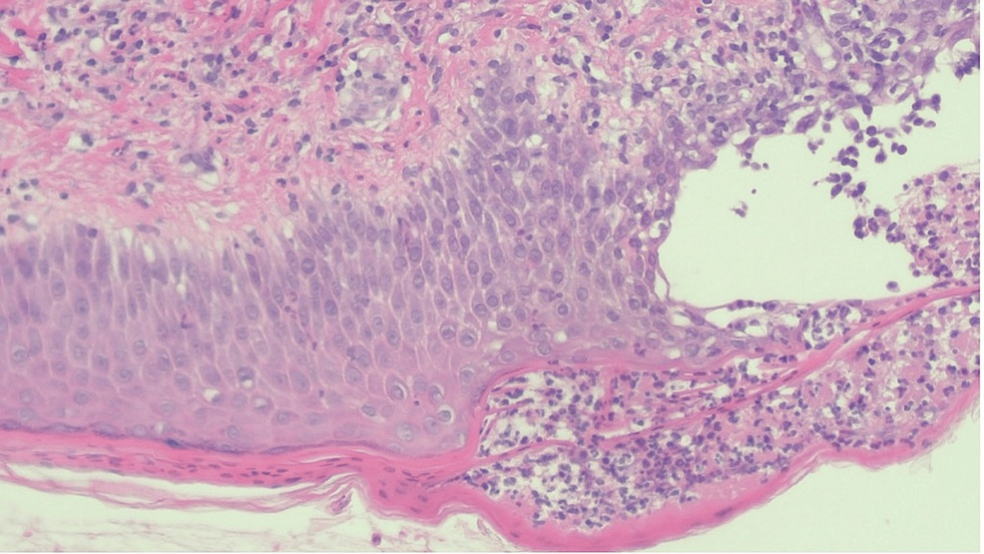
High-power view (20x) Histology slide at high power (20x) showing subcorneal and intraepidermal vesicles containing neutrophils, consistent with SSSS. SSSS: staphylococcal scalded skin syndrome.

The patient was pyrexial, and his C-reactive protein (CRP) was 87mg/L; however, his blood cultures remained negative. As the patient had an ongoing infection, it was decided he would not be for a second free flap at that time. The patient was brought back to the theatre for formal flap debridement and packing of the cavity. The Haematology team were consulted and advised that the thrombosis and subsequent flap failure may have been contributed to by a pro-thrombotic state induced by SSSS. There was no strong indication for intensive anticoagulation, and the patient remained on his prophylactic dose of 40mg of enoxaparin twice daily based on weight (103kg) for the duration of his admission. His rash and pain subsequently improved following 48 hours of antibiotics. Formal histological results showed subcorneal and intraepidermal vesicles containing neutrophils, consistent with SSSS (Figures 2, 3). His antibiotics were rationalised, and he completed 14 days of intravenous linezolid and clindamycin with a resolution of the rash.

In Figures 2, 3, punch biopsies from the left upper arm show a spongiotic dermatitis with underlying dermal oedema and mixed inflammation. The inflammatory infiltrate contains neutrophils, lymphocytes and rare eosinophils. Karyorrhectic debris is also present. There is no evidence of vasculitis. The high-power view shows a subcorneal, intraepidermal vesicle containing neutrophils. The appearances favour infection and would be consistent with the clinical impression of staphylococcal scalded skin syndrome.

In consultation with the patient and his family, it was decided that he would not be for another free flap during this admission. The patient was initially fitted with a temporary obturator as an inpatient and subsequently fitted with a custom-made obturator and zygomatic implants in consultation with the maxillofacial team. The patient was discharged after a total of 29 days of admission.

## Discussion

This is the first documented case of free flap failure associated with thrombosis from SSSS. Pre-operatively, the patient had no risk factors for flap failure. Intraoperatively, the patients vasculature was identified to be of good quality by experienced microsurgeons. The flap was monitored regularly as per standard protocol, by experienced ICU staff and members of the surgical team, and flap congestion was promptly noticed and acted on. However, despite best efforts, flap failure was the ultimate result.

SSSS usually commences as a localised skin infection with *S. aureus* strain, which produces an exfoliative toxin which spreads haematogenously causing epidermal damage at distant sites [10]. In this patient, the excoriated patch of eczema on his right hand was the likely source of infection. Given its haematogenous spread, *S. aureus* is usually identified in blood cultures in adult SSSS compared to paediatric SSSS [11]. This patient had been on Co-amoxiclav peri- and post-operatively, and this may explain his negative blood cultures. This toxin-mediated process causes T lymphocyte proliferation and massive cytokine release [12]. We hypothesise that this resulted in a hypercoagulable state causing recurrent macro- and micro-vascular thrombosis in this patient’s free flap.

Thrombosis at the anastomosis site is one of the most common causes of free flap failure, with the majority of free flap failures occurring in the first 48 hours [13,14]. Factors leading to thrombosis include kinks of the pedicle, tight closure, external compression, oedema, haematomas and thrombophilias, although there is no high-quality evidence to support the latter [15]. The proposed cause of flap thrombosis in this case was potentially an SSSS-induced procoagulant state. The role hypercoagulability plays in free flap failure remains controversial, and hypercoagulability itself is not a contraindication to free flap procedures [3].

The types of hypercoagulability may influence the risk of thrombosis. In patients with a known inherited or acquired hypercoagulable state, an anticoagulation management plan can be considered prior to surgery. However, as in this case, often the first indication of hypercoagulability in these patients is vascular thrombosis in the free flap. There are currently no consensus guidelines available on anticoagulation in free flap surgery in hypercoagulable patients. Multiple regimens have been described, with heparin being the most commonly used agent [3]. The decision to continue anticoagulation post-discharge is also controversial and patient specific, with no associated high-quality data. In this case, the pro-thrombotic state was deemed acquired and the patient only remained on heparin while an inpatient. In cases where there is a hereditary inherited or acquired thrombophilia identified pre-operatively, post-operative anticoagulation may be considered, depending on the specific nature of the thrombophilia [3].

Much of the literature documenting free flap failure due to thrombosis is in the context of genetic predispositions to clotting; however, causality is uncertain. Factor V Leiden is the most common inherited thrombophilia, with a documented incidence in Europe of up to 15% [4]. Protein S and protein C deficiency have a higher relative risk of venous thromboembolism compared to Factor V Leiden thrombophilia [4]. However, this risk has not been explored in the context of microvascular anastomosis [16]. These risks can be further altered depending on the gene status of the patients. In patients with Factor V Leiden, heterozygotes have a relative risk of venous thromboembolism of three to 10, whereas homozygotes have a relative risk of up to 79 [17]. Furthermore, patients can often have more than one co-existing thrombophilia, which further increases their risk of a thrombotic event. The role of routine genetic testing for pro-thrombotic states as part of free flap surgery workup is not supported by high-quality data and is not recommended. Patients with a personal or family history of a strong thrombophilia may be considered for further investigation. However, true strong hereditary thrombophilia is rare. Laboratory tests for thrombophilias are costly, and negative results do not fully exclude hereditary thrombophilia or other biological risks related to thrombosis [18,19]. The decision to perform thrombophilia screening in patients still varies between institutions and is often led by the Haematology service or guided by local guidelines.

Acquired pro-thrombotic states in free flap surgery are much more difficult to predict and manage. The patient described here had no personal or family history of clotting disorders. The cause of his hypercoagulable state was hypothesised to be contributed to the systemic effects of SSSS. The presence of a systemic infection is a known risk factor for thrombosis [6,20]. The greatest window of thrombotic risk is when the infection is active [21]. A hypothesised key difference between infection-associated thrombosis and thrombosis due to other aetiologies is there is a stronger inflammation-mediated component caused by the presence of the pathogen and its products. This results in the activation of platelets and damaged endothelium, which results in fibrin deposition and thrombus formation. This process is referred to as ‘thrombo-inflammation’ [20].

Pro-thrombotic states due to acquired factors such as systemic infections as a cause of free flap failure are a rare phenomenon. The COVID-19 pandemic did bring some attention to this idea, with a number of case reports of free flap failure in patients with COVID-19 reported [8,9]. COVID-19 is associated with both arterial and venous thrombus and has been suggested as an independent risk factor for free flap failure [19]. The exact mechanism of thrombosis in these patients is similar to that hypothesised in our patient, with the development of thrombo-inflammation in the context of an infective pathogen. However, due to the rarity of these presentations, there is still no formal guidance on anti-coagulation regimens. Based on observations from thrombosis in COVID-19 and free flap failure, Benmoussa et al. [8] advised on starting all patients with free flap failure and COVID-19 on at least 15 days of heparin. This advice was further supported by Al-Benna et al. in their reply to this article as heparin has both anti-coagulant and anti-inflammatory effects [22]. Heparin as both an anti-coagulant and anti-inflammatory agent in treating infection-induced thrombosis supports the hypothesis that thrombosis is the result of inflammatory immune dysregulation. Of note, the patient in this case had no recent history of COVID-19 pre-operatively. As mentioned, for our patient, anti-coagulation therapy was maintained for the duration of his inpatient stay resulting in a total of 29 days of treatment. Table 1 summarises the case reports of free flap failure due to the vascular effects of infection. Cases were identified by a PubMed search up to October 2023. Keyword searches included free flap + failure ± infection ± provoked ± thrombophilia.

**Table 1:** Summary of published cases documenting free flap failure associated with thrombotic effects of infection ALT: anterolateral thigh, VAC: vacuum-assisted closure, SSC: squamous cell carcinoma, RFF: radial forearm flap.

Author/Year	No. Cases	Defect	Flap	Infection	Arterial/Venous	Time to Thrombosis	Salvaged	Final Outcome
Chakraborty et al. [9] 2023	1	Traumatic right-side foot and ankle defect with extensor tendon and ankle joint exposure	ALT	COVID-19	Dermal network vessels	D8	Yes	VAC therapy and coverage with split-thickness skin graft
Zahran et al. [23] 2022	2	SCC involving left floor of mouth and extrinsic tongue musculature SCC involving floor of mouth and tongue	A. RFF B. ALT	Thrombophlebitis secondary to fistula	Arterial and venous	D11, D23	No	No further reconstruction. Reconstruction with a left pectoralis major myocutaneous flap and split-thickness skin graft

The limited number of documented cases (Table 1) highlights the rarity of this phenomenon. From the analysis of these cases, it appears that infection-induced flap failure follows a different pattern from the typical thrombo-embolic events seen in flap failure. Firstly, time to flap failure is much later than the usual 48-hour window with thrombosis [7,8]. Furthermore, infection induced followed a different pattern in each case. In Chakraborty et al. case [9], there was no fulminant free flap failure but necrosis of the skin from thrombosis of the dermal network in the context of COVID-19. Zahran et al.’s flap failures are a result of thrombophlebitis [23].

Venous thromboembolism (VTE) prediction tools are commonly used when prescribing anti-coagulation in surgical patients. Most institutions, including our own, have their own proforma which looks at risks such as immobility, malignancy, previous history of VTE, etc. The Caprini [24] and Barbar [25] are VTE risk assessment tools that were initially developed in medical cohorts but have since been validated in surgical patients [26]. Although validated in predicting general VTE risk in free tissue transfer patients [26,27], their ability to predict microvascular venous thrombosis in free flap reconstruction is poor. Two retrospective reviews looked at their use in predicting microvascular thrombosis in lower limb free flap reconstruction [28,29]. Both found no significant association between VTE risk and free flap failure following free tissue reconstruction of the lower extremities. Geoghegan et al. noted how all their patients who underwent free tissue transfer were deemed ‘high risk’ of developing deep vein thrombosis (DVT) according to these tools at baseline [30], resulting in an inability to further stratify those who would develop microvascular thrombosis and those who would not. However, the use of risk stratification tools is an area of potential development in free flap surgery. With National Flap registries, there are now large databases available that contain surgeon, patient and peri-operative information that may be used to create future microvascular thrombosis risk assessment tools.

## Conclusions

Venous thrombosis and free flap failure are devastating complications of microvascular free flap surgery. Hypercoagulable states either genetic or acquired should always be considered as a differential. This is the first documented case of free flap failure associated with SSSS. The management of this patient was complex and required a multi-disciplinary approach with input from Dermatology, Haematology, Surgeons and Intensivists. The presence of a systemic infection is a known risk factor for thrombosis through the thromboinflammation pathway. There is currently no concrete guidance on the management of hypercoagulable states in free flap surgery, with free flap failure often being the first sign of coagulopathy. The development of these types of guidelines is limited by the heterogeneity of causes and resulting cases. In this case, the decision to postpone a further free flap was patient specific and guided by the progressing clinical picture and risk of further thrombosis. Analysis of cases of free flap failure in the context of infection showed significant heterogeneity but suggests a different pattern of free flap failure compared to thrombosis due to ‘traditional’ causes.
